# Supplementary material for: Variation in the pelvic and pectoral girdles of Australian Oligo–Miocene mekosuchine crocodiles with implications for locomotion and habitus
Source: PeerJ. 2017 Jun 30;5:e3501. doi: 10.7717/peerj.3501 (PMC5494174; doi:10.7717/peerj.3501)
Supplement: Appendix S1 — Characters used in parsimony and bootstrap analysis of the pelvic materials. Characters 2,6, 8 are partially modified from characters 28 and 34 of Brochu (1999). [file peerj-05-3501-s001.docx]

Selected pelvic characters.

Characters used in parsimony and bootstrap analysis of the pelvic materials. Characters 2,6, 8 are partially modified from characters 28 and 34 of Brochu (1999).

(1) Ratio of the dorsoventral height of the ischial peduncle to the dorsoventral height of the acetabulum: less than 0.4 (0); equal or greater than 0.4 (1).

(2; 28 of Brochu (1999)) Ratio of the dorsoventral height of the postacetabular process to the dorsoventral height of the acetabulum: less than 0.6 (0); equal or greater than 0.6 (1).

(3) Anterior expansion of the pubic peduncle : absent (0); present (1).

(4) Pronounced asymmetry between the anterior and posterior condyles of the pubic peduncle: absent (0); present (1).

(5) Iliac margin of the acetabular perforation: well defined (0); weakly defined (1).

(6) Concavity of the acetabular space: shallow (0); deep (1).

(7; 28 of Brochu (1999)) Shape of the dorsal margin of the iliac blade: no indentation (0); moderate indentation (1); strong indentation (2).

(8) Orientation of the attachment for the M. ilioischio caudalis: posterior (0); dorsoposterior (1).

(9) Pronounced development of the ventral margin of the postacetabular process: absent (0); present (1).

(10; 34 of Brochu (1999)) Iliac crest forming anterodorsal prominence: absent (0); prominent (1); prominent encroaching anteriorly (2).

(11) Attachment sites of the transverse processes of the sacral vertebrae: centrally situated on the medial face of the ilium (0); ventrally situated on the medial face of the ilium (1).

(12) Pronounced convexity of the dorsal margin of the iliac crest: absent (0); present (1).

(13) Lateral expansion of the iliac crest: absent (0); weak (1); strong (2).
