# Supplementary material for: Variation in the pelvic and pectoral girdles of Australian Oligo–Miocene mekosuchine crocodiles with implications for locomotion and habitus
Source: PeerJ. 2017 Jun 30;5:e3501. doi: 10.7717/peerj.3501 (PMC5494174; doi:10.7717/peerj.3501)
Supplement: Appendix S2 — Characters scored according to the criteria detailed in Appendix S1 for each specimen and the pelvis of Gavialis gangeticus. Characters were unweighted and multistate characters were left unordered. [file peerj-05-3501-s002.docx]

Character-taxon matrix used for parsimony analysis.

Characters scored according to the criteria detailed in appendix 1 for each specimen and the pelvis of *Gavialis gangeticus*. Characters were unweighted and multistate characters were left unordered.

Taxon 10 13

*Gavialis gangeticus* 1111000002000

QM F57908 0100000001000

QM F57909 01---0000-000

NMV P228164 1100002011010

NMV P228635 11---0201-010

NMV P252361 -----0201-010

NMV P228627 -----02011010

QM F40581 110000-011010

QM F41198 1011111112001

QM F57911 1-1111---20--

NTM P908-35 1011111112001

NTM P891-5 101111---2001

NTM P5895 10---1111-001

QM F57913 10---12111102

QM F31406 10---12111102
